# Asia-Pacific ICEMR: Understanding Malaria Transmission to Accelerate Malaria Elimination in the Asia Pacific Region

**DOI:** 10.4269/ajtmh.21-1336

**Published:** 2022-10-13

**Authors:** Ivo Mueller, Amelie Vantaux, Stephan Karl, Moses Laman, Benoit Witkowski, Anais Pepey, Rebecca Vinit, Michael White, Alyssa Barry, James G. Beeson, Leanne J. Robinson

**Affiliations:** ^1^ Population Health & Immunity Division, Walter + Eliza Hall Institutes, Melbourne, Australia;; ^2^ University of Melbourne, Melbourne, Australia;; ^3^ Institute Pasteur Cambodia, Phnom Penh, Cambodia;; ^4^ Australian Institute of Tropical Health & Medicine, James Cook University, Cairns, Australia;; ^5^ PNG Institute of Medical Research, Madang, Papua New Guinea;; ^6^ Institut Pasteur, Paris, France;; ^7^ Deakin University, Geelong, Australia;; ^8^ Burnet Institute, Melbourne, Australia;; ^9^ Monash University, Victoria, Australia

## Abstract

Gaining an in-depth understanding of malaria transmission requires integrated, multifaceted research approaches. The Asia-Pacific International Center of Excellence in Malaria Research (ICEMR) is applying specifically developed molecular and immunological assays, in-depth entomological assessments, and advanced statistical and mathematical modeling approaches to a rich series of longitudinal cohort and cross-sectional studies in Papua New Guinea and Cambodia. This is revealing both the essential contribution of forest-based transmission and the particular challenges posed by *Plasmodium vivax* to malaria elimination in Cambodia. In Papua New Guinea, these studies document the complex host–vector–parasite interactions that are underlying both the stunning reductions in malaria burden from 2006 to 2014 and the significant resurgence in transmission in 2016 to 2018. Here we describe the novel analytical, surveillance, molecular, and immunological tools that are being applied in our ongoing Asia-Pacific ICEMR research program.

## INTRODUCTION

In 2014 and 2015, after a decade of significant progress in reducing malaria transmission, 21 Asia-Pacific countries signed up to the joint goal of regional malaria elimination by 2030 and established the Asia-Pacific Leaders Malaria Alliance (www.aplma.org) to monitor, support, and sustain their progress. Since then, progress has been mixed, with several countries reaching elimination and others reducing local transmission to very low levels. In other countries, progress has slowed or reversed, even before the COVID-19 pandemic put additional stresses on local public health systems.

Cambodia and Papua New Guinea (PNG) are two countries that exemplify the Asia-Pacific malaria elimination challenges. After the confirmation of artemisinin resistant *Plasmodium falciparum* in Western Cambodia in 2010 and its subsequent spread throughout the Mekong region,^1^^,^^2^ a major international effort to eliminate resistant *P. falciparum* malaria was launched. After an initial great reduction in *P. falciparum* and, to a lesser extent, *Plasmodium vivax* burden, gains slowed or in some parts were even reversed in 2017–2018 (reported malaria cases in 2010: 353,294, 2016: 124,137, 2018: 272,272^3^). The downward trend, however, recommenced and even dramatically accelerated in 2019 to 2021, with Cambodia reporting only 3,504 clinical malaria cases for 2021. *P. vivax* now accounts for ∼90% of Cambodian malaria cases.^4^ This indicates that Cambodia is close to eliminating local *P. falciparum* transmission. In PNG, after several long-lasting insecticidal net (LLIN) distribution rounds, the prevalence of *Plasmodium *sp. infections and number of confirmed malaria cases were reduced by 50% to 90% between 2008–2009 (prevalence from national malaria indicator survey [MIS]: 11.1%) and 2014 (MIS: 0.9%).^5^^–^^7^ However, cases resurged significantly between 2015 and 2017 (MIS: 6.2%)^8^ and have remained relatively high since then. Whereas transmission intensities of *P. vivax* and *P. falciparum* are comparable in PNG, *P. falciparum* still accounts for a clear majority of clinical cases.^3^

**Figure 1.**
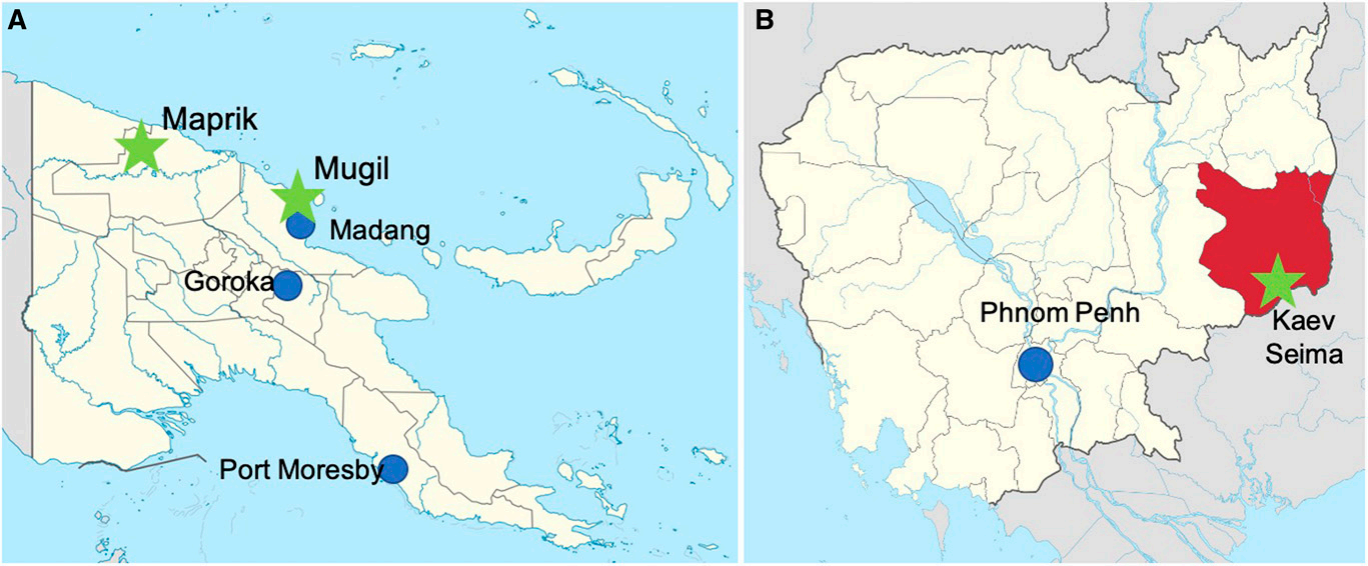
Locations of Asia-Pacific International Center of Excellence in Malaria Research field and laboratory sites. (**A**) Papua New Guinea (PNG): field sites (green stars) in Mugil, Madang Province, and Maprik, East Sepik Province, with PNG Institute of Medical Research laboratory sites (blue circles) in Madang (including insectary), Goroka and Port Moresby. (**B**) Cambodia: field site and insectary in Kaev Seima (green star), Modulkiri Province, and Institut Pasteur Cambodia laboratories in Phnom Penh. Internal boundaries delineate provinces.

Cambodia and PNG therefore constitute two distinct settings (Table 1) that exemplify the great diversity of malaria ecology in the Western Pacific region and the challenges countries face in the quest for malaria elimination. To better understand the reasons behind these divergent trends and highly dynamic patterns of *P. falciparum* and *P. vivax* in PNG and Cambodia, the Asia-Pacific and previous South-West Pacific International Center of Excellence in Malaria Research (ICEMR) have been conducting coordinated sets of epidemiological and entomological studies in three sites (Figure 1) with distinct malaria epidemiology, linked with in-depth laboratory studies to define host and parasite factors that contribute to sustaining ongoing malaria transmission, despite intensified control.

**Table 1:** Key characteristics of current Malaria eco-epidemiology in Cambodia and Papua New Guinea

	Cambodia	Papua New Guinea
Terrain	Dominated by large river flood plains of the Mekong and Tonle Sap River. Malaria transmission restricted to mountainous, highly forested areas along the Eastern, Western and Northern border areas.	Encompasses the Eastern half of New Guinea and offshore Islands. Main mountainous and remote areas. Malaria endemic in all areas below 1600 m altitude.
Climate/seasonality	Tropical monsoon climate with a wet season in May–October. Year-round transmission with more marked seasonality for *P. falciparum* than *P. vivax*.	Tropical monsoon climate with a wet season in December–April. Year-round transmission with modest seasonality in most areas. Areas above 1600 m are too cool for malaria transmission.
*Plasmodium* species	*P. falciparum*, *P. vivax*, *P. malariae*, *P. ovale*, and *P. knowlesi*	*P. falciparum*, *P. vivax*, *P. malariae*, and *P. ovale*
	*P. vivax* is the predominant source of infection and disease. *P. malariae* and *P. ovale* are rare, and current transmission is uncertain. *P. knowlesi* has been identified locally but transmission likely to be very low.	Comparable transmission of *P. falciparum and P. vivax* but *P. falciparum *is the dominant source of clinical illness except in children < 2 years. *P. malariae *and *P. ovale* infections are present but now relatively rare. No *P. knowlesi* transmission.
Key vector *Anopheles* species	Main vectors: *An. dirus *s.l., *An. minums*. Several scondary vectors important, include *An. barbirostris *s.l., *An. hycranus*.	Main vectors: *An. punctulatus* complex. Several minor vectors present.
	Outdoor biting, mostly in forest setting.	Both outdoor and indoor biting. Human and animal blood meals.
Transmission system	Forest malaria with largely occupational exposure	Peri-domestic transmission
High-risk groups	Forest-goers and mobile populations. Highest risk in adolescent and adult males.	Highest risk of malaria in children and pregnant mothers. Vivax is a disease of young children.

To facilitate this, the Asia Pacific ICEMR has developed a suite of novel analytical, surveillance, molecular, and immunological tools that are being applied in our ongoing program of coordinated epidemiological and entomological studies to accurately identify and delineate pockets of residual transmission in space and time.

### Investigating the extent and nature of spatial and temporal heterogeneity of malaria transmission.

In PNG, serial cross-sectional household surveys have been conducted in two malaria-endemic sites in Mugil on the coast north of Madang and in the Maprik area of East Sepik Province since 2005 (Figure 1), using sensitive molecular tools to determine the impact that intensified vector control and improved diagnosis and treatment have had on the epidemiology of *Plasmodium *spp. infections and transmission potential.^9^^–^^11^ We observed a considerable burden of low-density asymptomatic infections in these communities, which currently escape routine detection and treatment at health facilities. We observe a different impact on the prevalence of infection at the two sites and between the two dominant species. A high spatiotemporal variation in the risk of *Plasmodium* infection was identified in young 1- to 5-year-old children in 2013.^9^ More recently, generalized additive modeling was used to characterize the spatial heterogeneity of malaria risk in two villages on the north coast of Madang in 2014 and 2016 and to investigate the contribution of individual and household-level risk factors to malaria infection in these villages.^12^ Hotspots for *P. falciparum* were more commonly observed than for *P. vivax*. In the village of Megiar, some of the observed spatial risk could be explained by household risk factors—in particular, households that use outdoor surface water as their water source, presumably acting as a proxy for outdoor transmission.

In Cambodia, residual malaria is concentrated in remote, often poorly accessible border areas where transmission is associated with forest-related activities, such as logging or gem mining.^13^^–^^16^ As a consequence, the highest rates of malaria are observed in the adolescent and adult males who most engage in these activities. The ICEMR field site in Kaev Seima in the eastern province of Mondulkiri (Figure 1) fits this general pattern well. Epidemiological and semi-quantitative polymerase chain reaction (PCR) data from the 2018 community cross-sectional study revealed a prevalence of *Plasmodium *spp. infections of 8.3%, with 68% due to *P. vivax* and 96% of infections asymptomatic and/or undetectable by standard microscopy or rapid diagnostic tests.^17^ Prevalence ranged widely across villages, with very high levels of infections in villages inside the forest where all inhabitants were at risk of malaria infection. In villages outside the forest or on the fringe, the risk of infection was highly associated with work in and travel to the forest and highest in working-age men.

## USING IN-DEPTH IMMUNOLOGICAL AND GENOMIC STUDIES TO UNDERSTAND PARASITE–HOST INTERACTIONS

Despite notable differences in the epidemiology of malaria in PNG and Cambodia, there are also similarities. Asymptomatic infections of *P. falciparum* and *P. vivax* are highly prevalent in both countries, implying that a significant proportion of the populations at risk of malaria develops a substantial of immunity to malaria.

After repeated exposure to malaria, naturally acquired immunity develops, reducing the risk of severe and symptomatic illness, primarily by controlling blood-stage replication and thus limiting blood-stage parasite density.^18^^,^^19^ Acquired immunity is less effective in preventing infection per se, and asymptomatic blood-stage infections are often observed in individuals who have acquired significant immunity.^19^^,^^20^

Understanding the targets and functions of antibodies that provide protection against symptomatic malaria has been a major focus of our ICEMR program. We evaluated antibodies to a large number of merozoite antigens in longitudinal cohort studies using a combination of high-throughput ELISA and suspension bead array assays and identified antigen-specific IgG responses associated with protection from malaria due to *P. falciparum* and *P. vivax*.^21^^–^^24^ These antigens are potential targets of protective immunity, and our results can be compared with findings from related studies in other regions.^25^ This knowledge will be valuable to prioritize antigens as potential vaccine candidates. Antibodies to specific antigens or combinations may also be useful as biomarkers of exposure and immunity in population studies.^26^

Antibodies to some merozoite antigens can directly inhibit invasion of red blood cells (RBCs) to prevent blood-stage replication.^27^ Although this is a plausible mechanism of immunity, invasion or growth-inhibitory antibodies have not consistently or strongly correlated with protection, suggesting that other mechanisms are additionally important.^27^^,^^28^ A major focus of our ICEMR research is understanding immune mechanisms mediated by antibody fragment crystallizable (Fc)-regions, including complement fixation and engagement of Fc-receptors to promote phagocytosis and cellular cytotoxicity.

In studies of *P .falciparum* clinical immunity, we found that antibodies to merozoites fix and activate complement to inhibit invasion and lyse merozoites, and complement-fixing antibodies were more strongly correlated with protection than growth-inhibitory antibodies.^29^^,^^30^ Subsequently, we quantified complement-fixing antibodies to multiple merozoite antigens and identified antigen-specific complement-fixing antibodies (e.g. EBA140, MSP7, RALP1, GAMA, RH2, MSP-DBL1) that had strong associations with protection from symptomatic malaria or high density parasitemia (> 5,000 parasites/µL).^30^ Analysis of combinations of antigen-specific responses found that protective associations increased substantially up to combinations of three antigens and increasing the combination size to four to six antigens gave only minor improvements in the strength of protective associations. Antigens most frequently included in combinations with the highest protective associations included include RALP1, MSP7, Ripr, EBA140, PfRH2, and MSP-DBL1. Antibodies to surface antigens on *P. falciparum*–infected erythrocytes also contribute to immunity, especially in young children in our study populations, and antibodies predominantly target *P. falciparum* erythrocyte membrane protein 1.^31^^–^^33^

Studying functional immunity to *P. vivax* presents additional challenges, one of which is the severely limited capacity to propagate parasites in vitro. To get around this, we have optimized assays to quantify antibody complement-fixation and FcγR-mediated functions using arrays of *P. vivax* recombinant antigens.^34^ These approaches are applied to longitudinal cohorts to identify targets of functional Abs and specific responses, or response combinations, associated with protective clinical immunity. To obtain a complete picture of immunity in our study populations, we are also investigating the phenotypes and functions of neutrophils and monocytes, the major cell types mediating phagocytosis of parasites and contributing to malaria immunity^35^^–^^37^

Importantly, we and others have found that acquired immunity against *P. falciparum* and *P. vivax* is predominantly associated with prevention of clinical illness, rather than prevention of infection.^19^^–^^22^^,^^38^^–^^40^ Therefore, our data support the role of acquired immunity in maintaining asymptomatic infections that can sustain transmission.

However, the role of immunity in maintaining asymptomatic infections in populations with low malaria transmission such as in Cambodia is less clear because the current paradigm is that effective acquired immunity requires substantial malaria exposure to develop and requires regular exposure to be maintained. Some populations have transitioned from high to low transmission intensity. Therefore, ongoing protective immunity may be partly provided by established memory from prior exposures. However, there are other populations where low transmission has been established for an extended time, with lower magnitude of acquired Abs, yet low-density asymptomatic infections are still commonly observed.^41^^,^^42^ Ongoing studies seek to understand the basis for this. It may be that key immune responses can be acquired rapidly after limited malaria exposure and acquisition of immunity may be higher in adults than children; understanding this may provide important insights to inform vaccine development.^43^ Additionally, in settings with highly heterogeneous transmission, there may be subpopulations with greater exposure and acquisition of immunity.

To map how changes in transmission affect parasite populations and investigate whether the genetic composition of parasite population may contribute to high asymptomatic carriage, the Asia Pacific ICEMR Genomics Core has developed genotyping assays for malaria parasites using amplicon sequencing. These include 1) short single nucleotide polymorphism (SNP) microhaplotype assays for *P. falciparum*^44^^,^^45^ and *P. vivax* (Rosado et al., in preparation) to track clones in longitudinal cohort studies; 2) SNP barcodes: for *P. falciparum* (“GeoCode,” Harrison et al., in preparation) and *P. vivax*^46^ comprising more than 150 biallelic SNPs for measuring population genetic structure and the relatedness among malaria parasites; and 3) novel software tools for identifying AmpSeq haplotypes (HaplotypR,^47^ for population genetic analysis [VaxPack^48^] and evaluating SNP candidates in whole genome screens [PlasmoCaVaLieR, unpublished]).

AmpSeq markers for both *P. falciparum* and *P. vivax* have demonstrated high levels of diversity in PNG parasite populations, but low diversity in Cambodia, which reflects the lower transmission intensity and a largely clonal multidrug resistant *P. falciparum* population in Cambodia. A lower level of parasite diversity may enable protective immunity to be acquired more quickly, and studies are ongoing to understand this. In PNG ICEMR sites in Madang and East Sepik, parasite diversity was measured using 10 microsatellite markers but did not reveal any substantial changes, even with a large reduction in transmission intensity. However, an increase in multilocus disequilibrium, a measure of parasite relatedness, was observed suggesting this parameter may be a marker of transmission reduction. Contrary to expectations, *P. falciparum* population structure present at the first ICEMR timepoint in 2005–2006 was lost after LLINs were introduced and was attributed to increased human migration between the two ICEMR sites, whereas *P. vivax* remained unstructured both pre- and post-LLIN.^49^ Although small numbers of hypervariable microsatellites showed only subtle changes in parasite population structure, barcodes of at least 100 genome-wide SNPs with a much higher resolution identified strong bottlenecks and an increase in relatedness over time as transmission decreased. As transmission resurged, common *P. falciparum* lineages remained while *P. vivax* rapidly diversified (unpublished). This difference in results for the different marker panels is likely to be due to the SNPs more accurately characterizing parasite relatedness, and therefore changes in population structure. ICEMR studies are now utilizing these barcodes in PNG and Cambodia (Asia Pacific ICEMR), in addition to Mali (West Africa ICEMR). Whole genome sequencing is also being conducted in the three countries to validate SNP barcodes and confirm patterns observed.

Using ICEMR samples to monitor the evolution of antimalarial drug resistance mutations is a major goal of the program. Recently, *kelch13* C580Y mutations associated with artemisinin were discovered for the first time in PNG by genotyping *P. falciparum* isolates collected from Wewak, East Sepik Province in 2016.^50^ Although the genomic data were consistent with *kelch 13* C580Y emerging in the local parasite population, it was not clear whether it emerged in PNG, West Papua, or another nearby location. We recently developed a multiplex amplicon sequencing assay known as “DResCode” that targets well-known drug resistance genes (*kelch13*,* crt*,* mdr1*,* dhfr*,* dhps*,* mrp1*) in addition to a PNG genetic background that is associated with the *kelch13* C580Y mutation. Using this assay as well as a real-time quantitative PCR assay for rapid detection of *kelch13* C580Y mutations, ICEMR samples are being studied in a wider surveillance effort to confirm whether the *kelch13* C580Y mutation had spread to other parts of PNG.

Although the ICEMR site of Maprik is only 50 km from Wewak, where the first mutations were detected, no mutations were found among more than 300 samples collected in 2016. Genotyping of more than 600 samples collected from eight locations in PNG between 2015 and 2018, identified only a single C580Y mutant in Lae, Morobe Province.^51^ Ongoing work involves drug resistance genotyping of ICEMR samples using the DResCode assay as well as whole genome sequencing of a subset of these samples to establish the origins and spread of drug resistance in PNG and Cambodia. Prior studies have reported associations between acquired immunity and artemisinin resistance in the southeast Asia region, including Cambodia.^52^ Because host immunity contributes to parasite clearance, it can confound the assessment of drug resistance, and lower levels of malaria immunity may favor the evolution of resistance. Understanding the potential relationship between immunity and drug resistance in PNG is an important future goal.

## CHARACTERIZING LOCAL MALARIA TRANSMISSION SYSTEMS THROUGH IN-DEPTH STUDIES OF MOSQUITO VECTOR ECOLOGY, BEHAVIOR, AND RISK FACTORS FOR HUMAN-VECTOR CONTACT

Despite that spending extended periods of time in the forest is one of the main malaria risk factors in the Greater Mekong Subregion, few studies have collected *Anopheles* mosquitoes on forest fringes and in forest sites.^53^^–^^55^ Classically, malaria entomological surveys collect *Anopheles* vectors in villages from 6 pm to 6 am. To obtain a better knowledge of mosquito behavior in the Cambodian forest setting, we used odor-baited double nets traps to assess *Anopheles* behavior and host preferences over a 24-hour period in different ecological settings.^56^ Twenty percent of *Anopheles* vectors were captured during daytime, when people are active and not protected by a bed net, highlighting the importance of daytime biting to sustaining residual transmission in this area. Infectious mosquitoes (sporozoite positive in heads and thoraces) were collected in both human- and cow-baited double net traps, and key *Anopheles* vectors (e.g., *An. dirus *s.l.,* An. barbirostris *s.l.,* An. hyrcanus *s.l.) exhibited mainly generalist or zoophilic host preferences. These observations suggest that many of the *Anopheles* mosquito populations are largely maintained by feeding on animals, and that, in contrast to African malaria vectors, the notion of highly preferential human feeding may be inaccurate in the Greater Mekong Subregion. Knowing that opportunistic feeding is very common could inform the design of new vector control tools to control malaria infection levels.

We continue to investigate mosquito ecology by designing collection based on data acquired from land cover analyses and by conducting human mobility studies to better comprehend the transmission patterns. Our ICEMR is particularly interested in determining more finely the vector–human contact patterns in time and space. To do so, we assessed the evolution of the land cover over 30 years and showed a high deforestation rate with wooded areas decreasing from 91% to 47% in our study site.^57^ We developed GPS follow-ups of the local populations, which, with the land cover map produced, allows quantification of the different environments visited and the time spent therein. On the basis of these GPS follow-ups, we are now conducting mosquito vector collections in sites with high- and low-human visitation rates. Together, these approaches should allow a better understanding of forest-based malaria transmission in the Mekong area.

In the southwest Pacific, malaria transmission is known to be peri-domestic, and control relies heavily on the mass distribution of LLINs. Although there is no pyrethroid resistance in the Anopheline populations in PNG,^58^ the efficiency of LLINs to limit transmission is reduced by mosquito behaviors that are similar to that in the Cambodian setting, leading to less frequent contact with the nets. All relevant vectors in PNG are members of the *An. punctulatus* group, exhibiting opportunistic host-seeking behavior and a preference for outdoor biting. In addition, *An. farauti,* the dominant coastal vector species in PNG, is very active shortly after sunset. The effect of LLINs on these outdoor, zoophilic vector populations remains unclear. Our ICEMR has contributed significantly to the detailed understanding of these important vector ecology-related factors sustaining malaria transmission. By conducting targeted human landing catch studies indoors, outdoors, and in social gathering places, we quantified where transmission occurs. In addition, barrier screen studies revealed important host-seeking behaviors.

Although malaria prevalence in high-burden provinces in PNG is high on average, it is still characterized by a striking degree of spatiotemporal heterogeneity, resulting from human and mosquito-related factors. Our longitudinal mosquito collections revealed striking species-specific seasonality patterns in some settings, indicating that different species maintain malaria transmission through the wet and dry seasons, respectively (unpublished data). We also observed high differences in Anopheline biting frequency in villages that are just a few kilometers apart, indicating that high variability in local mosquito abundance is the main cause of transmission heterogeneity in PNG. A particular focus of our ICEMR lies on investigating when and where humans are most vulnerable to Anopheline biting and which population groups are most at risk. By matching human DNA collected in cross-sectional surveys with DNA obtained from blood-fed mosquitoes, our studies indicated that, as in other settings, younger males are at particular risk to receive mosquito bites, which is another parallel to the Cambodian setting, although the underlying reasons based on human occupational or behavioral patterns are different.^59^

Last but not least, we have established mosquito-to-human infection studies in PNG and Cambodia to investigate the contribution of different population groups (e.g., clinical versus asymptomatic) to malaria transmission.^60^^–^^62^ These studies confirmed the higher infectivity of high-density, symptomatic *P. falciparum* and *P. vivax* infections in both settings but also demonstrated the possibility of transmission from asymptomatic *P. vivax* infections. Further studies to determine the contribution of symptomatic versus asymptomatic infections to local transmission are ongoing.

By closely coordinating our entomology work with epidemiological studies, we are able to gain a holistic understanding of malaria transmission in both PNG and Cambodian communities.

## USING MATHEMATICAL MODELS TO PREDICT THE IMPACT OF MALARIA CONTROL INTERVENTIONS

Cross-sectional and longitudinal studies implemented in PNG and Cambodia provide a rich understanding of the epidemiology of *P. falciparum* and *P. vivax*.^17^ Analyses of infection prevalence and the incidence of clinical malaria are complemented by entomological surveillance to provide insight into the entire transmission cycle between humans and mosquitoes.^56^ Diverse epidemiological, clinical molecular, and serological data sets can be integrated into mathematical models, allowing for simulation of malaria transmission under differing scenarios.^63^^,^^64^ A key use of models is to predict how malaria epidemiology may change in response to control interventions.

In PNG, a model of *P. vivax* transmission was calibrated to malaria prevalence data collected after the introduction of insecticide treated nets.^65^ This model predicted a rebound in *P. vivax* cases if bed net distributions were stopped—a prediction consistent with most epidemiologists’ intuition. However, the model provided additional insight, predicting a rebound of malaria cases to levels even higher than before interventions. This is because children born during an era of high bed net coverage grow up with limited malaria exposure and consequently have low levels of immunity. If bed net distribution stops, this cohort is extremely vulnerable to new malaria cases. These findings from mathematical models add to the evidence base needed to justify continuing bed net distribution.

In Cambodia, age and gender stratified analysis of data from cross-sectional surveys revealed important covariates for malaria risk.^17^ Within forest villages, high levels of *P. vivax* prevalence were observed (23%), and infections were distributed across men and women of all ages. Outside the forest, *P. vivax* prevalence was lower (3%) with significant clustering of infections in men aged 16 to 50 years, consistent with a pattern of occupational exposure where working-age men are exposed to malaria during forest visits. This is in contrast to the “free-mixing” assumption of many malaria transmission models, where any mosquito can bite any human. These epidemiological findings have initiated theoretical model development to account for the structured interactions between humans and mosquitoes.^66^ Ongoing studies aim to integrate data on population exposure and immunity to malaria generated using serological tools.

## CONCLUSIONS

The Asia-Pacific ICEMR’s ability to bring together long-term epidemiological and entomological follow-up with state-of-the-art laboratory assays and analytical tools has provided unique insights into the complex host–parasite–vector interactions that underlie the different trajectories and challenges of malaria control and elimination in PNG and Cambodia.

Our Cambodian results strengthen the hypothesis that forests are the main risk areas for human malaria transmission and highlight the importance of daytime biting behavior as a potential source for transmission. Approaches targeted at risk groups based only on forest proximity may be more cost-effective for the national malaria control program. Our results also highlight the importance of specific control efforts aimed at asymptomatic *P. vivax* infections. This will require both the implementation of effective radical cure with Primaquine and the development of novel interventions, such as serological testing and treatment (PvSeroTAT) that allow identifying and targeting potential hypnozoite carriers. In PNG, the ICEMR not only provided a detailed understanding of the key factors that have driven both the decrease in transmission between 2010 and 2014 but also for the resurgence after 2015. By linking the continuous monitoring of the key threats of increasing drug resistance and variations in vector control efficacy, our studies are uniquely placed to support the PNG national malaria control program in policy and implementation. Studies of the targets and mechanisms of malaria immunity, including diversity of key target antigens, will inform the development of vaccines for *P. falciparum* and *P. vivax* to help achieve long-term control and elimination of malaria in the Asia-Pacific region, and elsewhere.
